# Effect of Lead on the Transformation of Urea-N in Fly Ash-Contaminated Soils under Different Moisture Regimes

**DOI:** 10.1100/tsw.2007.129

**Published:** 2007-06-12

**Authors:** D. K. Das, P. Bashetti, S. K. Naik, Pintu Sur

**Affiliations:** ^1^ Department of Agricultural Chemistry and Soil Science, Bidhan Chandra Krishi Viswavidyalaya, Mohanpur-741252, Nadia, West Bengal, India; ^2^ National Research Centre for Orchids, Pakyong-737106, Sikkim, India

**Keywords:** lead, moisture regime, NH_4_-N, NO, urea-N, transformation

## Abstract

Urea is applied to soil for plant growth and undergoes various changes while in the soil. The mobility of heavy metals changes with the transformation of urea applied to the soil that, in turn, affects the activity of microbial biomass. The objective of this study was to determine the interaction between lead (Pb) and urea under two moisture regimes (60% and saturated water contents). At 60% water content, the amount of NH_4_-N decreased with the incubation time, irrespective of Pb and urea applied, while the amount of NO_3_-N content in the soil gradually increased with the incubation period to 28 days, and such an increase was counteracted by application of Pb. The amount of NO_3_-N content was higher than that of NH_4_-N, however, under saturated moisture condition, the amount of NH_4_-N was increased with the incubation time regardless of the levels of Pb application. The amount of NO_3_-N content in the soil gradually decreased with the advance of incubation at saturated condition. Although application of urea increased the NO_3_-N content in the soil, such an increase was suppressed by the application of different levels of Pb at water saturated condition throughout the incubation period.

### INTRODUCTION

The soils of high traffic density areas and other industrial regions have been polluted from a wide range of sources with Pb, Cd, Hg, As, and other heavy metals. With the expansion of industrialization in India, the problem of the disposal of industrial effluents, in both liquid and solid forms, has been drawing the attention of many soil scientists and environmentalists. These pollutants reach agricultural lands directly or indirectly[1]. Soderstrom et al.[2] reported that with decreased soil water potential, following mineral N application and declining pH resulting from nitrification of $NH_4^+$ sources, the activity of microorganisms is reduced. However, application of fertilizer can directly stimulate the growth of microbial populations as a whole by applying nutrients and may affect the composition of individual microbial communities in soil[3,4].

The added heavy metals degrade the soil by modifying the ionic composition and biological activity. Urea, extensively used as a source of N, is applied to the soil. Urea undergoes many changes before the N becomes available to the plants[5]. Pollutants, such as sewage-sludge and other industrial effluents, are disposed to the environment and contain heavy metals, including Pb. When urea is added to the soils contaminated with heavy metals, especially with Pb, transformation of urea may influence the mobility of the heavy metals. The rate of urea hydrolysis was not a linear function of moisture content, but increased with field capacity, and the added urea was completely hydrolyzed within 3 days at 40, 80, and 100% field capacity[6]. Antil et al.[7] suggested that the urea hydrolysis followed the first-order kinetics and the rate of hydrolysis reduced with an increase in the Pb content of the soil. The objective of the present investigation was to study the interaction between Pb and urea under two distinct moisture regimes (60% and saturated water contents).

### EXPERIMENTAL METHODS

The soil sample (0–15 cm depth) was collected from the Tribeni area of West Bengal, India, where the soil was contaminated mainly by fly ash. The soil sample was then air dried, powdered, and passed through an 80-mesh sieve. The physicochemical properties of the soil are given in Table 1. The composition of the contaminated fly ash was analyzed separately and the total contents were Fe 600, Mn 450, Cu 48, Zn 210, Ni 148, and Pb 18 mg $kg^{-1}$. Ten grams of soil were taken in a series of incubation tubes. Pb at 0, 10, 20, and 50 mg $kg^{-1}$ prepared from lead chloride ($PbCl_2$) and N at 0, 50, 100, and 200 mg $kg^-1$ prepared from urea in the form of solution were added to the soil. The experiment was laid down in completely randomized design (CRD) with factorial concept. The 16 treatment combinations were replicated thrice. Analysis of variance (ANOVA) was carried out by CRD. The least significant difference (LSD) test was applied to evaluate the significance of the differences between the individual treatments.

**TABLE 1**
**Physicochemical Properties of the Experimental Soil**

| Properties | Particulars |
|---|---|
| pH | 6.48 |
| EC ($dSm^{-1}$) | 0.26 |
| Organic C (g $kg^{-1}$) | 14.8 |
| CEC[C mol ($p^+$) $kg^{-1}$] | 18.5 |
| Available N (kg $ha^{-1}$) | 464.0 |
| Available P (kg $ha^{-1}$) | 283.2 |
| Exchangeable K (kg $ha^{-1}$) | 360.2 |
| DTPA extractable Pb (mg $kg^{-1}$) | 7.6 |

Two moisture regimes, i.e., one at 60% water holding capacity (WHC) of soil and the other at saturated condition, were maintained in all the samples throughout the incubation time by applying double distilled water every alternate day. All treatments were kept in the laboratory at a room temperature of about 30 ± 1°C. Then the soils were incubated for 0, 7, 14, 21, and 28 days under the two moisture regimes. At the end of the experiment, the soils were analyzed for the DTPA extractable Pb, following the method of Lindsay and Norvell[8], using an atomic absorption spectrophotometer (Perkin Elmer model A Analyst 100). The $NH_4$-N and $NO_3$-N contents were determined using the method described by Sahrawat and Burford[9].

## RESULTS AND DISCUSSION

### Transformation of $NH_4$-N at 60%WHC

The results show that the amount of $NH_4$-N content in the soil was significantly decreased with the progress of incubation, irrespective of Pb and urea added (Table 2). The magnitude of such a decrease varied with the treatments under 60% WHC. The $NH_4$-N content in the soil recorded highest at 7 days after incubation and thereafter gradually decreased to 28 days at all levels of Pb and urea applied. The magnitude of the decrease was highest at 28 days after incubation. The amount of $NH_4$-N content significantly increased over control following urea application and such an increase was counteracted by the application of different levels of Pb. Gupta and Chaudhry[10] observed that low temperature along with Pb delayed the hydrolysis of urea significantly and the $NH_4$-N persisted for a longer period in the soil. The magnitude of decrease in $NH_4$-N content was highest with higher levels of Pb and urea applications. Nitant[11] reported that the amount of $NH_4$-N content increased with increasing levels of urea while that of the same content decreased with the application of Pb. The $NH_4$-N content decreased with the progress of incubation and the maximum decrease was recorded in $Pb_{20}N_0$ and $Pb_{50}N_0$ treatment combinations throughout the incubation period.

**TABLE 2**
**Effect of Different Levels of Pb and N on the Changes in $NH_4$-N (mg $kg^{-1}$) Content in Soil under 60% WHC**

| Treat-ments | Period of Incubation (Days) | | | | | | | | | | | | | | | |
|---|---|---|---|---|---|---|---|---|---|---|---|---|---|---|---|---|
| | 7 | | | | 14 | | | | 21 | | | | 28 | | | |
| | $N_0$ | $N_{50}$ | $N_{100}$ | $N_{200}$ | $N_0$ | $N_{50}$ | $N_{100}$ | $N_{200}$ | $N_0$ | $N_{50}$ | $N_{100}$ | $N_{200}$ | $N_0$ | $N_{50}$ | $N_{100}$ | $N_{200}$ |
| $Pb_0$ | 26.4 | 40.3 | 50.7 | 78.9 | 23.4 | 36.7 | 43.7 | 61.2 | 17.2 | 22.4 | 25.8 | 41.9 | 12.0 | 15.6 | 14.9 | 27.8 |
| $Pb_{10}$ | 24.9 | 38.4 | 53.6 | 76.5 | 23.4 | 29.8 | 41.9 | 60.2 | 17.1 | 21.6 | 27.8 | 50.8 | 12.1 | 15.5 | 13.7 | 37.6 |
| $Pb_{20}$ | 21.6 | 40.8 | 50.0 | 70.9 | 23.2 | 28.6 | 33.7 | 49.8 | 17.0 | 20.9 | 24.9 | 37.6 | 11.8 | 16.3 | 14.2 | 29.6 |
| $Pb_{50}$ | 23.1 | 45.6 | 52.0 | 69.4 | 22.8 | 32.6 | 35.7 | 50.6 | 16.8 | 23.7 | 26.2 | 38.0 | 11.9 | 15.9 | 18.8 | 23.2 |
| LSD ($p$ = 0.05) | | | | | | | | | | | | | | | | |
| Pb | | | 1.89 | | | | 2.15 | | | | 1.77 | | | | 1.70 | |

The highest percent decrease (18.1%) of $NH_4$-N content was recorded in $Pb_{20}N_0$ treatment combination, suggesting a suppressive effect of Pb on the $NH_4$-N content in the soil resulting through the very little production of $NH_4$-N from the urea hydrolysis. However, such a decrease in $NH_4$-N was due to the slower rate of ammonification throughout the incubation period as affected by the application of various levels of Pb[12].

Fig. 1 shows the mean effect of Pb on the $NH_4$-N content in the soil under 60% WHC. The $NH_4$-N content gradually decreased with increased application of Pb up to 14 days of incubation and thereafter showed inconsistent decrease. It was observed that the highest decrease in $NH_4$-N content was found when Pb was applied at 20 mg $kg^{-1}$ throughout the incubation period. The percent decrease in $NH_4$-N content from 7 to 28 days of incubation was 64.2, 59.2, 57.04, and 63.23% at 0, 10, 20, and 50 mg $kg^{-1}$ Pb, respectively. The mean effect of urea application on the changes in $NH_4$-N content in the soil under 60% WHC is given in Fig. 2. The $NH_4$-N content increased significantly with higher levels of N and gradually decreased with the progress of the incubation period. The percent decrease from 7 to 28 days after incubation was 50.2, 61.7, 70.13, and 60% at 0, 50, 100, and 200 mg $kg^{-1}$ N, respectively.

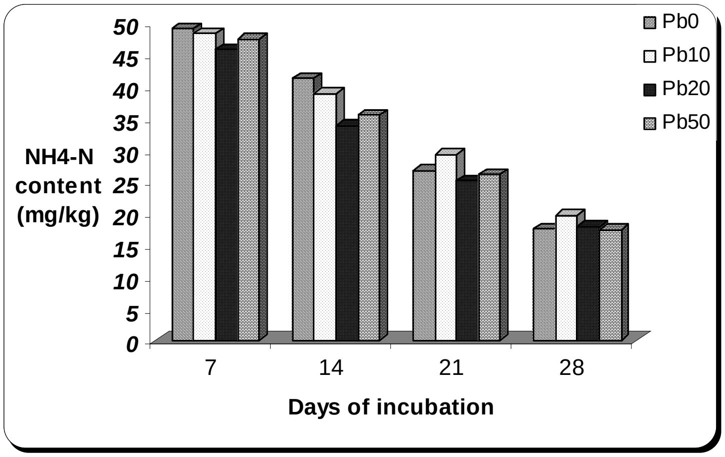

**FIGURE 1**. Mean effect of different levels of Pb on the changes in $NH_4$-N content in soil under 60% WHC.

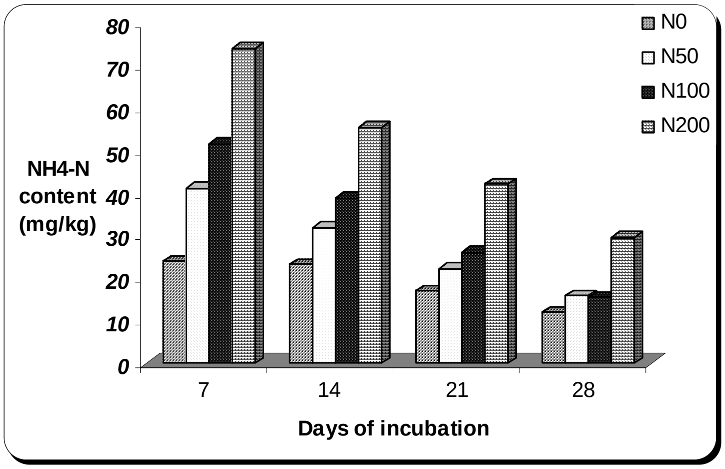

**FIGURE 2**. Mean effect of different levels of N on the changes in $NH_4$-N content in soil under 60% WHC.

### Transformation of $NO_3$-N at 60%WHC

The amount of $NO_3$-N content in the soil at 60% WHC was gradually increased with the progress of the incubation period of 28 days, irrespective of Pb and urea applied (Table 3). The magnitude of increase, however, was found to be significantly changed with the treatments. The highest amount of $NO_3$-N content of 140.8 mg $kg^{-1}$ was recorded in the treatment $Pb_0N_{200}$ (where 0 and 200 mg $kg^{-1}$ of Pb and N was applied), showing 306.9% increase over control ($Pb_0N_0$). Antil et al.[7] also observed that the amount of $NO_3$-N content enhanced with the incubation period and the maximum nitrification was observed between 7 and 14 days of incubation.

Increasing the levels of Pb (10–50 mg $kg^{-1}$) significantly decreased the $NO_3$-N content throughout the period of incubation, while increasing the levels of N (50 to 200 mg $kg^{-1}$) significantly increased the $NO_3$-N content. This is also supported by Gupta and Chaudhry[13]. Such an inhibitory effect of different levels of Pb may be due to the toxicity to the activity of nitrifiers[14,15]. The amounts of $NO_3$-N were found significant among the different treatments when Pb and N were applied at their higher level while the same was at par with different levels of Pb when no N was added.

**TABLE 3**
**Effect of Different Levels of Pb and N on the Changes in in $NO_3$-N (mg $kg^{-1}$) Content in Soil under 60% WHC**

| Treat-ments | Period of Incubation (Days) | | | | | | | | | | | | | | | |
|---|---|---|---|---|---|---|---|---|---|---|---|---|---|---|---|---|
| | 7 | | | | 14 | | | | 21 | | | | 28 | | | |
| | $N_0$ | $N_{50}$ | $N_{100}$ | $N_{200}$ | $N_0$ | $N_{50}$ | $N_{100}$ | $N_{200}$ | $N_0$ | $N_{50}$ | $N_{100}$ | $N_{200}$ | $N_0$ | $N_{50}$ | $N_{100}$ | $N_{200}$ |
| $Pb_0$ | 34.6 | 50.6 | 60.9 | 98.7 | 42.5 | 59.1 | 72.6 | 116.8 | 44.6 | 75.9 | 93.7 | 130.0 | 49.8 | 83.6 | 99.6 | 140.8 |
| $Pb_{10}$ | 34.0 | 46.2 | 63.9 | 90.2 | 41.8 | 59.0 | 78.4 | 100.6 | 43.5 | 69.6 | 94.6 | 108.4 | 49.5 | 76.2 | 104.5 | 117.0 |
| $Pb_{20}$ | 34.2 | 49.8 | 59.9 | 83.6 | 42.0 | 61.8 | 68.7 | 90.7 | 44.0 | 79.4 | 92.8 | 108.4 | 48.6 | 85.4 | 103.4 | 121.6 |
| $Pb_{50}$ | 33.5 | 56.1 | 63.1 | 79.6 | 41.3 | 69.4 | 76.9 | 91.8 | 43.7 | 84.0 | 89.8 | 100.6 | 48.2 | 94.0 | 98.3 | 111.2 |
| LSD ($p$ = 0.05) | | | | | | | | | | | | | | | | |
| Pb | | | 1.34 | | | | 1.77 | | | | 2.99 | | | | 1.73 | |
| N | | | 1.34 | | | | 1.77 | | | | 2.99 | | | | 1.73 | |
| Pb × N | | | 2.69 | | | | 3.53 | | | | 5.97 | | | | 3.45 | |

As can be seen in Fig. 3, the amount of $NO_3$-N content in soil at 60% WHC was gradually increased with the progress of the incubation period irrespective of treatments and decreased significantly with the successive addition of Pb throughout the incubation period. The percent increase from 7 to 28 days of incubation was 48.6, 48.2, 57.8, and 51.4% at 0, 10, 20, and 50 mg $kg^{-1}$ Pb, respectively. The mean effect of urea application (Fig. 4) on $NO_3$-N content showed that the amount of $NO_3$-N content gradually increased with the incubation time. The percent increase from 7 to 28 days of incubation was 36.54, 60.35, 63.68, and 39.34% at 0, 50, 100, and 200 mg $kg^{-1}$ N, respectively.

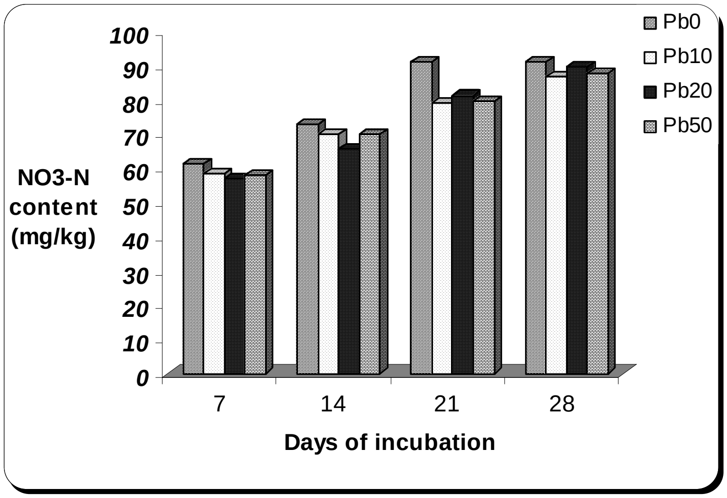

**FIGURE 3.** Mean effect of different levels of Pb on the changes in $NO_3$-N content in soil under 60% WHC.

### Transformation of $NH_4$-N at Saturated Moisture Condition

The amount of $NH_4$-N content gradually increased with the progress of the incubation period, irrespective of Pb and urea applied (Table 4). The magnitude of such an increase varied with the treatments, recording a significant increase with the application of N at 200 mg $kg^{-1}$ over that of the control. However, such an increase in $NH_4$-N content in soil at saturated condition decreased with the application of Pb at different

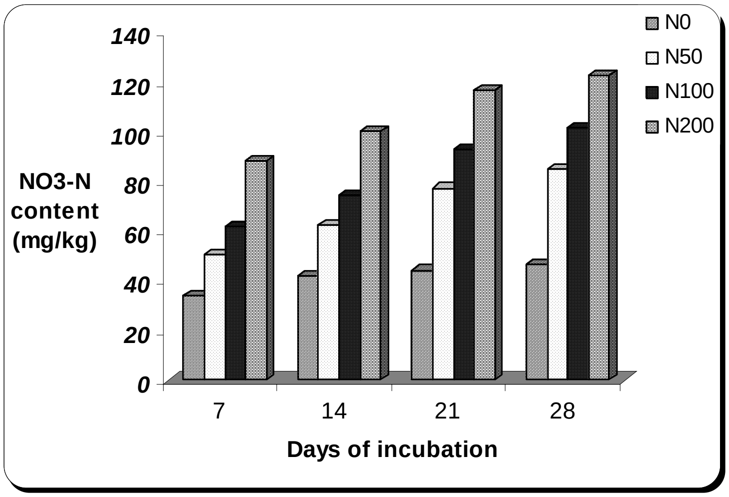

**FIGURE 4.** Mean effect of different levels of N on the changes in $NO_3$-N content in soil under 60% WHC.

**TABLE 4**
**Effect of Different Levels of Pb and N on the Changes in $NH_4$-N (mg $kg^{-1}$) Content in Soil under Saturated Condition**

| Treat-ments | Period of Incubation (Days) | | | | | | | | | | | | | | | |
|---|---|---|---|---|---|---|---|---|---|---|---|---|---|---|---|---|
| | 7 | | | | 14 | | | | 21 | | | | 28 | | | |
| | $N_0$ | $N_{50}$ | $N_{100}$ | $N_{200}$ | $N_0$ | $N_{50}$ | $N_{100}$ | $N_{200}$ | $N_0$ | $N_{50}$ | $N_{100}$ | $N_{200}$ | $N_0$ | $N_{50}$ | $N_{100}$ | $N_{200}$ |
| $Pb_0$ | 31.7 | 45.6 | 58.5 | 85.3 | 32.6 | 47.3 | 59.5 | 86.7 | 36.0 | 59.5 | 68.5 | 98.6 | 41.9 | 64.2 | 76.2 | 110.4 |
| $Pb_{10}$ | 30.0 | 43.4 | 60.2 | 83.6 | 32.4 | 49.8 | 67.2 | 99.9 | 43.6 | 60.6 | 72.2 | 106.2 | 50.1 | 72.2 | 79.8 | 118.0 |
| $Pb_{20}$ | 29.8 | 44.1 | 58.1 | 81.4 | 31.4 | 50.7 | 65.1 | 106 | 42.7 | 60.6 | 76.4 | 117.0 | 60.2 | 67.4 | 90.3 | 124.0 |
| $Pb_{50}$ | 29.4 | 49.7 | 58.6 | 79.8 | 30.3 | 56.7 | 70.0 | 93.0 | 41.8 | 69.1 | 81.1 | 107.0 | 28.7 | 76.8 | 85.0 | 122.0 |
| LSD ($p$ = 0.05) | | | | | | | | | | | | | | | | |
| Pb | | | 2.73 | | | | 1.03 | | | | 1.94 | | | | 1.47 | |
| N | | | 2.73 | | | | 1.03 | | | | 1.94 | | | | 1.47 | |
| Pb × N | | | 5.46 | | | | 2.06 | | | | 3.89 | | | | 2.94 | |

levels. The application of Pb in saturated soil might have some effect on the retardation of urea hydrolysis. The result of the present investigation was in agreement with the findings of Antil et al.[7] who revealed that the microbial biomass and urease activity declined with increasing levels of Pb in the soil. Urea hydrolysis was faster in uncontaminated soils (97.2% within 24 h) compared to contaminated soil (91.1% within 24 h). They also observed that the maximum accumulation of $NH_4$-N was found between 7 and 14 days in the soils containing 496.4 and 589.9 mg $kg^{-1}$ Pb, respectively.

Pal and Chhonkar[16] reported that the reduction of the urea hydrolysis rate may be ascribed to the activation of urea molecules by sodium and hydroxyl ions. The results also showed that the higher $NH_4$-N concentration was recovered in Pb-treated soils, as Pb likely caused $NH_4$-N persistence in the soil by restricting the activity of nitrobacters and a higher amount of $NH_4$-N was recorded with higher levels of Pb[13]. The persistence of a greater amount of $NH_4$-N content at the later period of incubation might be due to the very slower rate of urea hydrolysis under saturated soil moisture content, with higher levels of Pb and N application.

Fig. 5 illustrates that the mean effect of Pb did not show significant difference on $NH_4$-N content among different levels of Pb within the incubation period and higher $NH_4$-N content was recovered in Pb-treated soils throughout the incubation period. The maximum $NH_4$-N content was found when Pb was applied at 20 and 50 mg $kg^{-1}$ at later stages of incubation, as it tried to make $NH_4$-N persist, probably by restricting the activity of nitrobacters with higher levels of Pb. This is also supported by Gupta and Chaudhry[10] who reported that a higher concentration of Pb at 400 mg $kg^{-1}$ delayed the nitrification and $NH_4$-N persisted in the soil for a longer period. Fig. 6 summarizes the mean effect of urea application on the changes in $NH_4$-N content under saturated soil condition. The $NH_4$-N content increased significantly with higher levels of N and gradually increased with the progress of incubation time. The percent increase from 7 to 28 days of incubation was 74.45, 53.50, 40.73, and 43.72% at 0, 50, 100, and 200 mg $kg^{-1}$ N, respectively (Fig. 6).

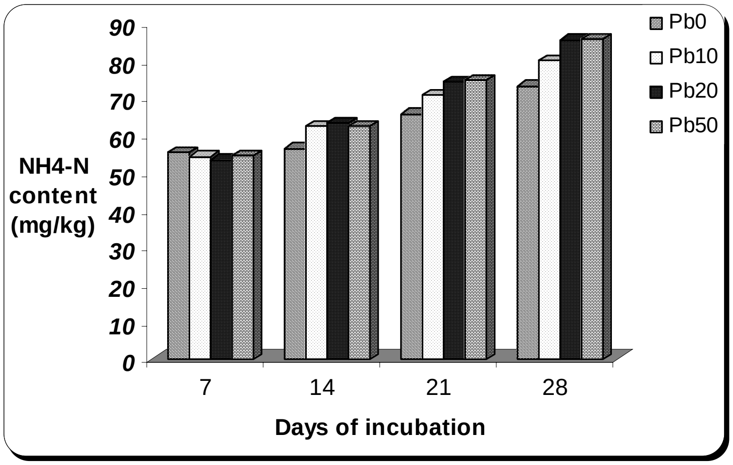

**FIGURE 5**. Mean effect of different levels of Pb on the changes in $NH_4$-N content under saturated soil.

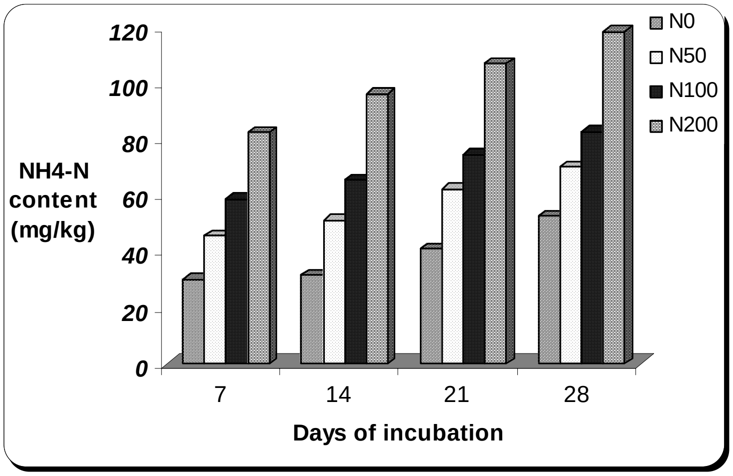

**FIGURE 6**. Mean effect of different levels of N on the changes in $NH_4$-N content under saturated soil.

### Transformation of $NO_3$-N at Saturated Moisture Condition

The changes in $NO_3$-N content in saturated soil gradually decreased with time, irrespective of the treatment (Table 5). The lowest $NO_3$-N content was recorded in control treatment. The application of urea, however, increased the amount of $NO_3$-N content in the soil initially and, thereafter, the amount of the same decreased at the later period of incubation. Such magnitude of variation has been found to be counteracted either by the sole application of Pb or the combined application of Pb and N at their various levels. Considering the interaction, it was observed that the amount of $NO_3$-N content significantly decreased in $Pb_{50}N_0$ treatment, which was closely followed by $Pb_{10}N_0$ and $Pb_{20}N_0$ treatments, suggesting an antagonistic effect between Pb and N in relation to changes in $NO_3$-N content in soils.

The amount of $NO_3$-N in saturated soil has always been found to be lower than $NH_4$-N. Such a decrease, however, was further enhanced with the application of Pb. Such a decrease might be due to the very slow rate of urease activity, as well as nitrification. Gupta and Chaudhry[13] reported that heavy metal decreases the rate of hydrolysis particularly at the high level of 400 mg $kg^{-1}$. The retardation was in the order of Hg > Zn > Ni > Pb. Urease activity is sensitive to different heavy metals as urease-SH groups react with metallic cations at higher concentrations.

**TABLE 5**
**Effect of Different Levels of Pb and N on the Changes in $NO_3$-N (mg $kg^{-1}$) Content in Soil under Saturated Condition**

| Treat-ments | Period of Incubation (Days) | | | | | | | | | | | | | | | |
|---|---|---|---|---|---|---|---|---|---|---|---|---|---|---|---|---|
| | 7 | | | | 14 | | | | 21 | | | | 28 | | | |
| | $N_0$ | $N_{50}$ | $N_{100}$ | $N_{200}$ | $N_0$ | $N_{50}$ | $N_{100}$ | $N_{200}$ | $N_0$ | $N_{50}$ | $N_{100}$ | $N_{200}$ | $N_0$ | $N_{50}$ | $N_{100}$ | $N_{200}$ |
| $Pb_0$ | 24.2 | 35.9 | 46.5 | 70.9 | 22.8 | 32.1 | 43.2 | 58.6 | 18.6 | 26.8 | 34.6 | 43.7 | 12.1 | 17.2 | 24.4 | 30.9 |
| $Pb_{10}$ | 23.8 | 35.8 | 47.6 | 71.1 | 21.6 | 30.0 | 40.8 | 56.3 | 15.8 | 24.6 | 30.7 | 41.2 | 11.7 | 15.6 | 19.3 | 26.7 |
| $Pb_{20}$ | 23.3 | 36.1 | 47.0 | 68.0 | 21.2 | 28.9 | 42.6 | 50.0 | 15.4 | 23.6 | 30.4 | 36.9 | 11.5 | 17.9 | 21.2 | 23.4 |
| $Pb_{50}$ | 22.7 | 35.3 | 46.0 | 68.4 | 18.7 | 26.6 | 35.3 | 54.2 | 14.7 | 21.4 | 25.6 | 41.6 | 11.2 | 17.6 | 19.7 | 28.4 |
| LSD ($p$ = 0.05) | | | | | | | | | | | | | | | | |
| Pb | | | 1.97 | | | | 1.18 | | | | 1.60 | | | | 2.55 | |
| N | | | 1.97 | | | | 1.18 | | | | 1.60 | | | | 2.55 | |
| Pb × N | | | 3.95 | | | | 2.36 | | | | 3.20 | | | | 5.10 | |

The research results also pointed out that the amount of $NO_3$-N content was lower in Pb-treated soil compared to control, which suggests that the rate of nitrification under saturated condition is slower in the presence of Pb, which also inhibits the urease activity[17]. Gupta and Chaudhry[13] also reported that the concentration of $NO_3$-N content in the control treatment was significantly higher than in the metal-treated soil. They also reported that increasing levels of Pb, Ni, Hg, and Zn from 200 to 400 mg $kg^{-1}$ soil $NO_3$-N content significantly decreased, which is in line with the results of our investigation.

As can be seen in Fig. 7, $NO_3$-N content significantly decreased with the increase of Pb concentrations (0–50 mg $kg^{-1}$). The maximum $NO_3$-N content was recorded at 7 days after incubation and gradually decreased with the progress of incubation. The percent decrease from 7 to 28 days after incubation was 52.1, 58.9, 57.5, and 55.4% at 0, 10, 20, and 50 mg $kg^{-1}$ Pb, respectively. Fig. 8 represents the mean effect of urea application on the changes in $NO_3$-N content at saturated condition. The $NO_3$-N content significantly increased with higher levels of N and the $NO_3$-N content gradually decreased with the progress of the incubation period. The percent decrease in $NO_3$-N content from 7 to 28 days after incubation was 50.55, 52.3, 54.77, and 60.7% at 0, 50, 100, and 200 mg $kg^{-1}$ N, respectively.

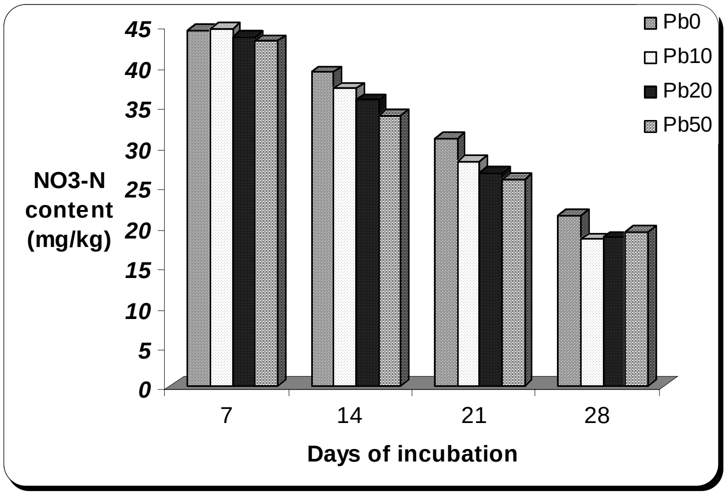

**FIGURE 7**. Mean effect of different levels of Pb on the changes in $NO_3$-N content under saturated soil.

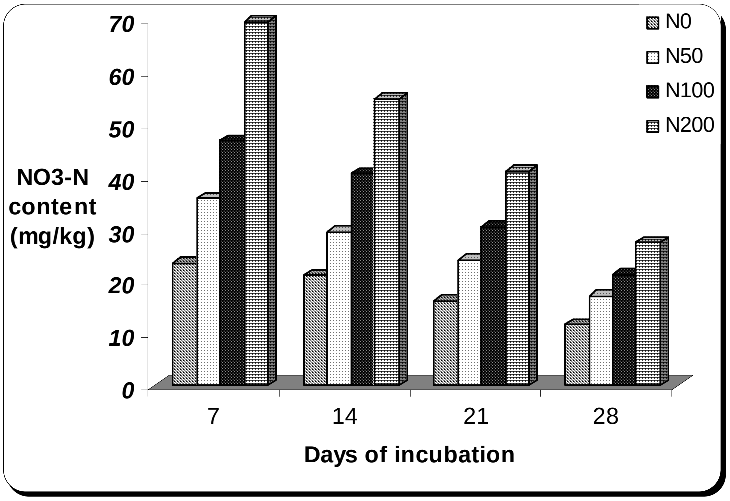

**FIGURE 8**. Mean effect of different levels of N on the changes in $NO_3$-N content under saturated soil.

### CONCLUSIONS

The present experimental results confirmed that the amount of $NO_3$-N at 60% WHC was always higher as compared to $NH_4$-N content, suggesting a higher rate of nitrification prevailing at 60% WHC than at saturated condition. Increasing the level of Pb decreases the $NO_3$-N and increases the $NH_4$-N content, which might be due to the moderation of ammonification-nitrification processes. The result suggested the existence of an antagonism effect between Pb and N in relation to changes in $NO_3$-N content in the soil.
